# Genome-Wide Association Study of Parasite Resistance to Gastrointestinal Nematodes in Corriedale Sheep

**DOI:** 10.3390/genes13091548

**Published:** 2022-08-27

**Authors:** Beatriz Carracelas, Elly A. Navajas, Brenda Vera, Gabriel Ciappesoni

**Affiliations:** 1 Department of Animal Breeding, Instituto Nacional de Investigación Agropecuaria, INIA Las Brujas, Ruta 48 Km 10, Canelones 90100, Uruguay; 2 National Research Program on Meat and Wool Production, Instituto Nacional de Investigación Agropecuaria, INIA Las Brujas, Ruta 48 Km 10, Canelones 90100, Uruguay

**Keywords:** FEC, genomic regions, ssGWAS

## Abstract

Selection of genetically resistant animals is one alternative to reduce the negative impact of gastrointestinal nematodes (GIN) on sheep production. The aim of this study was to identify genomic regions associated with GIN resistance in Corriedale sheep by single-step genome-wide association studies (ssGWAS) using 170, 507 and 50K single nucleotide polymorphisms (SNPs). Analysis included 19,547 lambs with faecal egg counts (FEC) records, a pedigree file of 40,056 animals and 454, 711 and 383 genotypes from 170, 507 and 50K SNPs, respectively. Genomic estimated breeding values (GEBV) were obtained with single-step genomic BLUP methodology (ssGBLUP), using a univariate animal model, which included contemporary group, type of birth and age of dam as class fixed effects and age at FEC recording as covariate. The SNP effects as wells as *p*-values were estimated with POSTGSF90 program. Significance level was defined by a chromosome-wise False Discovery Rate of 5%. Significant genomic regions were identified in chromosomes 1, 3, 12 and 19 with the 170 SNP set, in chromosomes 7, 12 and 24 using the 507 SNP chip and only in chromosome 7 with the 50K SNP chip. Candidate genes located in these regions, using Oar_v4.0 as reference genome, were *TIMP3*, *TLR5*, *LEPR* and *TLR9* (170 SNPs), *SYNDIG1L* and *MGRN1* (507 SNP chip) and *INO80*, *TLN2*, *TSHR* and *EIF2AK4* (50K SNP chip). These results validate genomic regions associated with FEC previously identified in Corriedale and other breeds and report new candidate regions for further investigation.

## 1. Introduction

Parasitic infections caused by gastrointestinal nematodes (GIN) are the diseases with the greatest impact upon health and productivity for grazing ruminants, mainly sheep and goats, because they can cause weight loss, poorer growth performance and even death [1]. In Uruguay, most prevalent parasites in sheep are *Haemonchus contortus* and *Trichostrongylus colubriformis* [2,3]. One strategy to reduce the negative impact of GIN is by selection of genetically resistant animals, because GIN resistance is a moderately heritable trait [4,5,6]. In Uruguay, since 1994, genetic resistance for GIN is included in the Corriedale National Genetic Evaluation using faecal egg counts (FEC) measured in lambs as selection criterion. This evaluation is conducted by the National Institute of Agricultural Research (INIA) and the Uruguayan Wool Secretariat (SUL). FEC is the main indicator of GIN resistance in sheep, with high correlation with adult parasite burden [7], and low-to-moderate heritabilities have been reported [8,9].

Identification of quantitative trait loci (QTL) associated with GIN resistance or susceptibility could improve the selection process and the understanding of biological processes related to host immune response [10]. Genome-wide association studies (GWAS) are used to identify genomic regions where candidate genes associated with a phenotypic trait are located. This methodology assumes that these genes are in linkage disequilibrium (LD) with a single nucleotide polymorphism (SNP), which means that these regions are physically connected and will segregate together in the population [11].

With single-step GBLUP (ssGBLUP), breeding values and marker effects can be estimated simultaneously, adjusting for population structure at the same time [12,13]. In addition, with this methodology non-genotyped animals in the pedigree and their phenotypes are also considered, without computing pseudo data [14]. Wang et al. [14] showed that SNP effects could be derived from the genomic estimated breeding values (GEBV) and later used for the single-step GWAS (ssGWAS), a methodology that has been validated in several species, such as bovine [15,16,17,18,19] and swine [20,21]. The advantage of ssGWAS compared to multi-step GWAS methods is that it integrates all pedigree, genomic and phenotypic information from genotyped as well as non-genotyped individuals in a one-step procedure, which allows the use of any model and all relationships simultaneously [14].

In sheep, GWAS have been extensively used to detect SNPs associated with economically important traits. The first GWAS in sheep was done to understand horns genetic basis in wild Soay sheep [22] and since then, multiple GWAS have reported QTL associated with growth, carcass and meat quality and reproductive traits [23]. Regarding GIN parasite resistance, Benavides et al. [24] reported 123 markers significantly associated with FEC and related traits for *H. contortus, Teladorsagia circumcincta* and *T. colubriformis* parasites. In another study, Berton et al. [25] found genomic regions associated with FEC using ssGWAS in Santa Inês sheep.

Periasamy et al. [26] identified 170 SNPs associated with 76 candidate genes involved in immune response to GIN resistance. In turn, INIA developed a 507 SNP chip with Affymetrix (Charrua custom array), that contains 174 SNPs related to FEC trait and 258 paternity SNPs [27,28].

The aim of this study was to identify genomic regions associated with GIN parasite resistance in Corriedale sheep using 170, 507 and 50K SNPs, applying the ssGWAS approach, and explore the biological processes underlying the genetic resistance. 

## 2. Materials and Methods

### 2.1. Phenotypic and Pedigree Data

From 2000 to 2019, FEC records were collected in autumn from 19,547 Corriedale lambs (8779 females and 10,768 males) belonging to 29 extensive production farms (24 stud flocks, three experimental units and two progeny testing centrals). Corriedale is a dual-purpose breed (wool and meat production), originally developed in New Zealand from crossing Lincoln or Leicester rams with Merino females [29], which represents 42% of the national sheep flock in Uruguay [30]. FEC sampling was performed according to the protocol used for genetic evaluations [31]. Briefly, lambs were treated with an anthelmintic at weaning and then FEC was monitored until a FEC average of 700 eggs per gram of faeces was reached, with no more than 20% of individuals with zero counts. At that point, all lambs were sampled, with faecal samples taken directly from the rectum and sent to the parasitology laboratories at INIA or SUL, where FEC were assessed using a modified McMaster technique with a sensitivity of 100 eggs per gram of faeces [32]. Due to FEC’s non-normal distribution, data were transformed to natural logarithm as described by Ciappesoni et al. [33]: LogFEC = Log_e_ (FEC + 100). Descriptive statistics are presented in Table 1.

Genealogical information from 40,056 animals was provided by the Rural Association of Uruguay and the Uruguayan Corriedale Breeders Society, who also supplied the FEC data recorded at the stud flocks. 

### 2.2. Genotypic Data

Genomic information from Corriedale animals with FEC phenotypes and complete pedigree information was available from three different sources, linked to different research initiatives. The three densities included here are: 170 SNPs (International Atomic Energy Agency) [26], a 507 SNP chip (Charrua custom array; Affymetrix, Santa Clara, CA, USA) [27,28] and a 50K SNP chip (Ovine SNP50 BeadChip v1 and v2, Illumina, San Diego, CA, USA; and Applied Biosystems™ Axiom™ Ovine Genotyping Array 50K, Thermo Fisher Scientific, Waltham, MA, USA). Mostly male and female lambs were selected for genotyping, and all sires that were available related to those lambs.

At the time our analysis was undertaken, the distribution of genotyped animals was as follows. A total of 289 lambs of both sexes, belonging to one experimental unit, born between 2000 and 2011, and 46 sires and 119 dams, were genotyped with 170 SNP. In the case of the 507 SNP chip, genotypes of 565 lambs of both sexes, belonging to one experimental unit, born between 2000 and 2011, and 35 sires and 111 dams, were available. Finally, 265 lambs of both sexes, born between 2000 and 2019, belonging to two experimental units, as well as 64 sires and 54 dams were genotyped with the 50K SNP chip.

All DNA samples were extracted, based on the protocol defined by Medrano et al. [34], from 1548 blood samples collected by jugular vein puncture using tubes with K2 EDTA anticoagulant (BD Vacutainer, Becton, Dickinson and Company, Franklin Lakes, NJ, USA). NanoDrop™ 8000 spectrophotometer (Thermo Fisher Scientific, Waltham, MA, USA) was used for DNA quantification and purity evaluation (260/280 ratio). DNA integrity was checked with a 1% agarose gel with a 0.5X TBE buffer (Tris-Borate-EDTA, Sigma-Aldrich, San Luis, MO, USA) during 25 min at 100 V. Finally, DNA samples were stored at −80 °C until genotyping was performed.

Genomic data quality control included the exclusion of markers located in sexual chromosomes, monomorphic SNPs and those with minor allele frequencies (MAF) < 0.05 and call rate < 90%; and animals with call rate < 90%. Numbers of SNPs and animals after quality control, used in this study, are presented in Table 2. Note that for the 50K SNP chip, only the 33,236 SNPs in common between the Illumina and Applied Biosystems platforms were considered, which explains the 29,832 SNPs in Table 2. 

**Figure 1:**
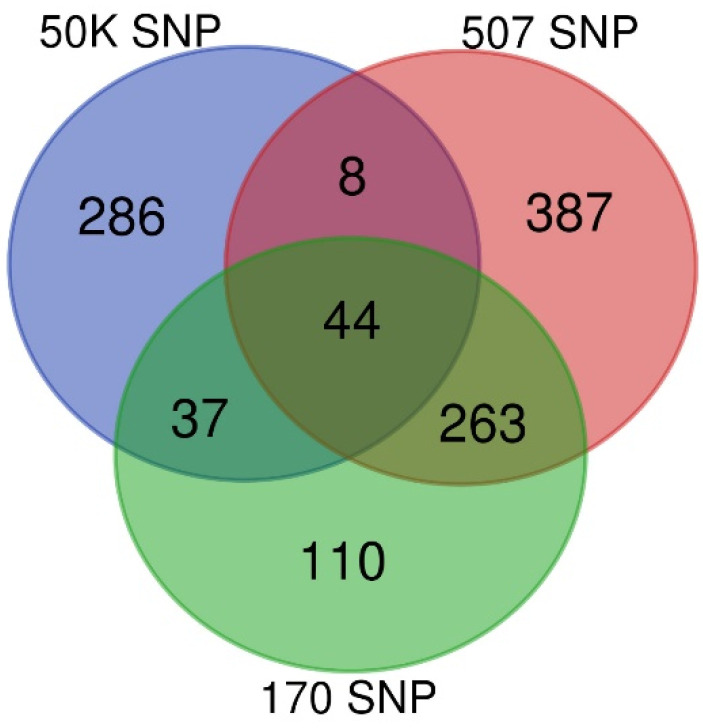
Number of individuals genotyped with 170, 507 and 50K SNPs.

Only the 507 SNP chip has 91 SNPs in common with the 50K SNP chip, with no SNPs overlapping among the other chips. Figure 1 shows the number of genotyped individuals with each SNP density, and the number of individuals genotyped with more than one chip.

Even though there were subsets of animals genotyped with two or three SNP chips (Figure 1), the number of animals in these subsets was limiting for GWAS. Preliminary analysis with the largest subset of 307 animals genotyped with both 170 and 507 SNPs did not show common significant loci.

### 2.3. Statistical Analysis

Association analysis for FEC was performed for each SNP density (170, 507 and 50K) using ssGWAS methodology [14] with BLUPF90 family of programs [35].

The GEBVs for FEC were estimated based on described phenotypic, pedigree and genomic information, based on the following univariate animal model:

(1) y=Xb+Zu+e 

where *y* is the vector of LogFEC; *b* is the vector of fixed effects, which includes 467 contemporary groups (birth year, gender, farm, and management flock), type of birth (two levels: unique or multiple), age of dam (three levels: 2, 3 and ≥4 years old) and age at FEC recording as covariate, *u* is the vector of additive genetic effects, with *u*~*N* (0, *H σ*^2^*_u_),* where *H* is the matrix of pedigree and genomic information, and *σ*^2^*_u_* is the additive genetic variance; *e* is the vector of random residuals *e*~*N* (0, *I σ*^2^*_e_)* being *I* the identity matrix and *σ*^2^*_e_* the residual variance; and *X* and *Z* are the incidence matrices of fixed and additive genetic effects, respectively. 

The inverse of this matrix was constructed according to Aguilar et al. [36]:

(2) $$H^{-1}=A^{-1}+\left(\begin{array}{cc} 0 & 0\\ 0 & G^{-1}-A_{22}^{-1} \end{array}\right)\;$$

where $A^{-1}$ is the inverse of the pedigree relationship matrix for all animals, $G^{-1}$ is the inverse of the genomic relationship matrix, and $A_{22}^{-1}$ is the inverse of the pedigree relationship matrix for genotyped animals. *G* matrix was computed according to VanRaden [37]:

(3) $$G={ZDZ}^{\prime }q\;$$

where *Z* is the matrix that relates genotypes of each locus adjusted for allelic frequencies, *D* is the diagonal matrix of weights for SNP variances (initially *D* = *I*) and *q* is a normalization factor. GEBV mean accuracies, standard deviations, minimum and maximum are reported as supplementary material (Table S2).

Briefly, SNP effects were derived with the following iterative process described by Wang et al. [14]:

1. Establish the diagonal matrix of weights for SNP variances as an identity matrix: *D = I*
2. Construct *G* matrix = *ZDZ′q*
3. Estimate GEBVs for all animals included in the pedigree using ssGBLUP
4. Convert GEBVs to SNP effects: $\hat{u}={qDZ}^{\prime }G^{*-1}{\hat{a}}_{g}$, where ${\hat{a}}_{g}$ is genotyped animals GEBV
5. Estimate each SNP weight (*i*): $d_{i}={\hat{u}}_{i}^{2}2p_{i}\left(1-p_{i}\right)$
6. Normalize the SNP weights for the total additive genetic variance to remain constant
7. Exit or return to step 3.

In the current study, the process included only one iteration because no changes were observed in marker effects when different weights were used

Each SNP *p*-value was calculated as described by Aguilar et al. [17]:

(4) $$P_{i}=P_{t}\left(\frac{{\hat{u}}_{i}}{\sqrt{{\hat{\sigma }}_{i}^{2}/n}},\;n-1\right)\;$$

where $P_{t}$ is the distribution function of the *t* distribution, ${\hat{u}}_{i}$ is the *i*th SNP effect, *n* is the number of animals with the *i*th SNP and ${\hat{\sigma }}_{i}^{2}$ is the *i*th SNP genetic variance. SNP effects and *p*-values were estimated with POSTGSF90 [38].

After obtaining the significance levels, Manhattan plots were generated with R software [39]), where *Ovis aries* autosomic chromosomes are represented with its −log_10_ (*p*-values). A quantile-quantile (QQ) plot was used to evaluate if population structure was accurately accounted for in the model [40]), since if this is not fulfilled it could result in false positive associations in the ssGWAS [41]. This type of plot compares the observed and expected by chance −log_10_ significance values of each SNP [42]. 

The most frequently used procedures to adjust for multiple testing are Bonferroni correction and False Discovery Rate (FDR) [43]. The FDR methodology is less conservative than Bonferroni correction [44] and allows deeper exploratory analysis. In addition, Misztal et al. [45] indicated that a Bonferroni correction is not recommended for GWAS in livestock since the extent of LD is much larger than in humans [46]. Preliminary studies showed that when Bonferroni correction was applied to our data, only one SNP was detected with the 170 SNPs. In the current study, significant SNPs were defined by using a chromosome-wise FDR of 5% using the p.adjust function in R. SNPs with a chromosome-wise FDR < 0.05 were selected to identify candidate genes for logFEC.

### 2.4. Functional Gene Annotation

Two databases were used for gene identification based on the Oar_v4.0 ovine reference genome: iSheep database [47] and Genome Data Viewer [48]. Candidate genes were considered as such if their boundaries fell within 20 kbp upstream or downstream of the significant SNPs [49]. The identification of proteins and gene ontology (GO) terms was performed using UniProt database [50]. These ontologies are structured vocabularies constructed by the Gene Ontology project, which are applied in the annotation of gene products in biological databases [51]. This project comprises three ontologies: molecular function, cellular component and biological process. In this study, genes related to biological processes and specifically to immune function were identified based on GO terms.

## 3. Results

### 3.1. GWAS

#### 3.1.1. Genome-Wide Associations Using 170 SNPs

**Figure 2:**
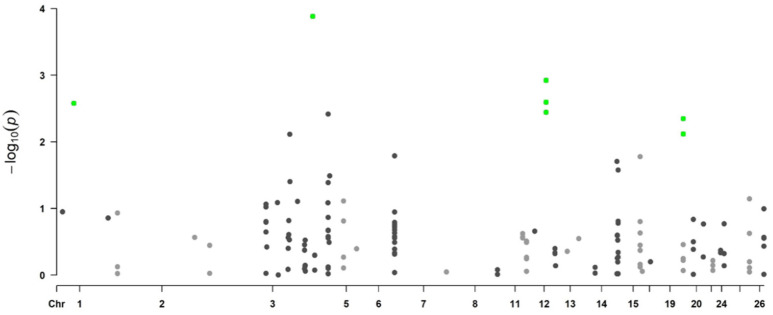
Manhattan plot for logFEC representing genome-wide associations using 170 SNPs and 454 genotyped individuals. *Y*-axis shows each SNP -log_10_ (*p*-value) and *x*-axis shows the SNP position across the 26 chromosomes. Green dots represent the significant SNPs using a chromosome-wise FDR of 5%, black and gray dots are non-significant SNPs.

**Figure 3:**
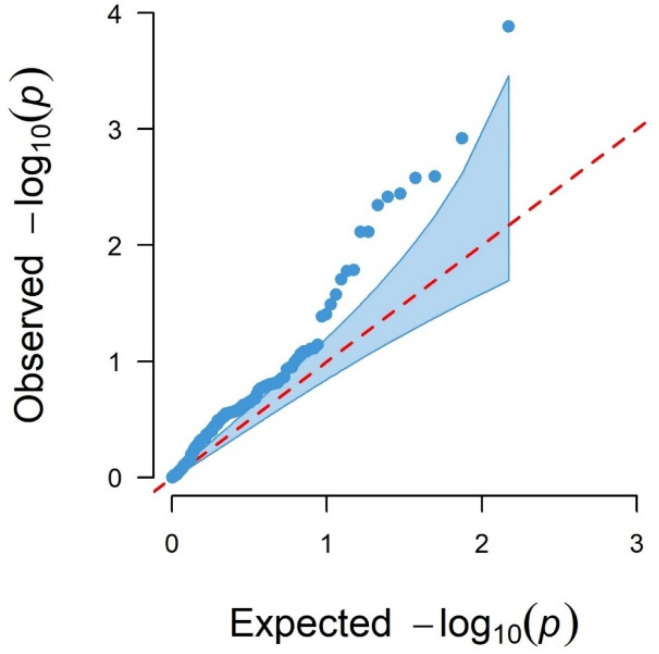
QQ plot of observed −log_10_ (*p*-values) (blue dots) and expected −log_10_ (*p*-values) (red dashed line) from GWAS results using the 170 SNPs.

Figure 2 shows the Manhattan plot for logFEC using 170 SNPs and its corresponding QQ plot (Figure 3). In this case, 16 SNPs with *p*-values < 0.05 were identified, seven of them significant with the 5% FDR per chromosome, which were located in chromosomes 1, 3, 12 and 19 (Table 3). The QQ plot shows that most *p*-values follow a uniform distribution (straight line), but these seven SNPs observed *p*-values deviate from the expected ones, suggesting an association between them and logFEC (Figure 3).

In OAR3, one significant SNP is within the *TIMP3* gene (*metallopeptidase inhibitor 3*), and another SNP is in OAR1 in the *LEPR* gene (*leptin receptor*). Another three SNPs in OAR12 are located within the *TLR5* gene (*toll-like receptor 5*) and two other SNPs in OAR19 are within the *TLR9* gene region (*toll-like receptor 9*).

#### 3.1.2. Genome-Wide Associations Using the 507 SNP Chip

**Figure 4:**
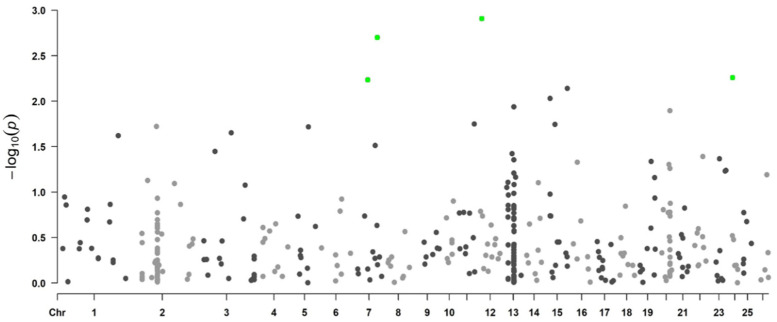
Manhattan plot representing genome-wide associations with logFEC using the 507 SNP chip and 702 genotyped individuals. *Y*-axis shows each SNP −log_10_ (*p*-value) and *x*-axis shows the SNP position across the 26 chromosomes. Green dots represent the significant SNPs using a chromosome-wise FDR of 5%, black and gray dots are non-significant SNPs.

**Figure 5:**
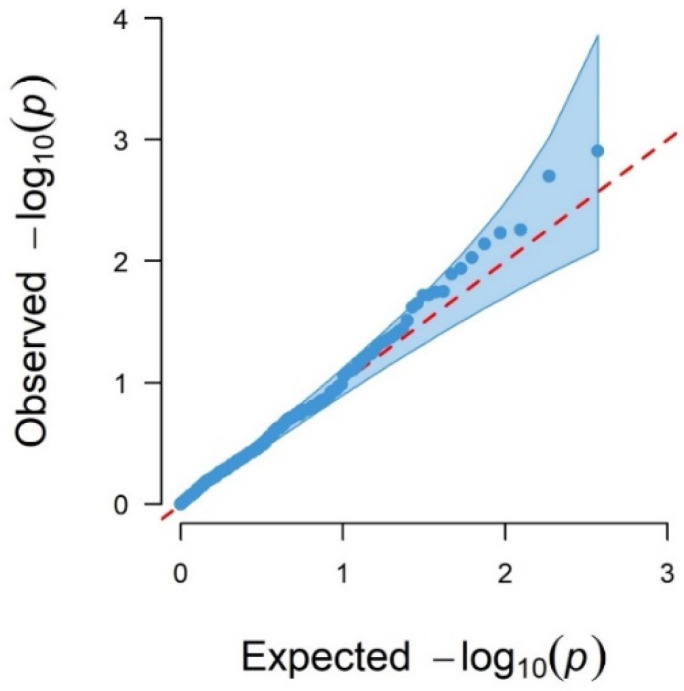
QQ plot of observed −log_10_ (*p*-values) (blue dots) and expected −log_10_ (*p*-values) (red dashed line) from GWAS results using the 507 SNP chip.

Figure 4 and Figure 5 show the Manhattan plot for logFEC using the 507 SNP chip and its corresponding QQ plot, respectively. In this case, 22 SNPs were identified with *p*-values < 0.05, and from these, four SNPs were significant with the 5% FDR per chromosome. These SNPs are located in chromosomes 7, 12 and 24 (Table 4) and were originally obtained from the 50K SNP chip when the 507 SNP chip was developed. The QQ plot shows that most *p*-values follow a uniform distribution (straight line). An association between these four SNPs and logFEC is suggested by the deviation of observed *p*-values from the expected ones (Figure 5).

In OAR24, one significant SNP is within the *MGRN1* gene (*mahogunin ring finger 1*) and another SNP in OAR7 is close to the *SYNDIG1L* gene (*synapse differentiation inducing 1-like*).

#### 3.1.3. Genome-Wide Associations Using the 50K SNP Chip

**Figure 6:**
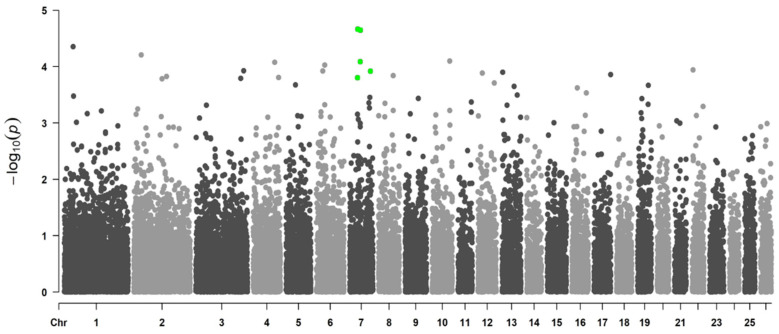
Manhattan plot representing genome-wide associations with logFEC using the 50K SNP chip and 375 genotyped individuals. *Y*-axis shows each SNP -log_10_ (*p*-value) and *x*-axis shows the SNP position across the 26 chromosomes. Green dots represent the significant SNPs using a chromosome-wise FDR of 5%, black and gray dots are non-significant SNPs.

**Figure 7:**
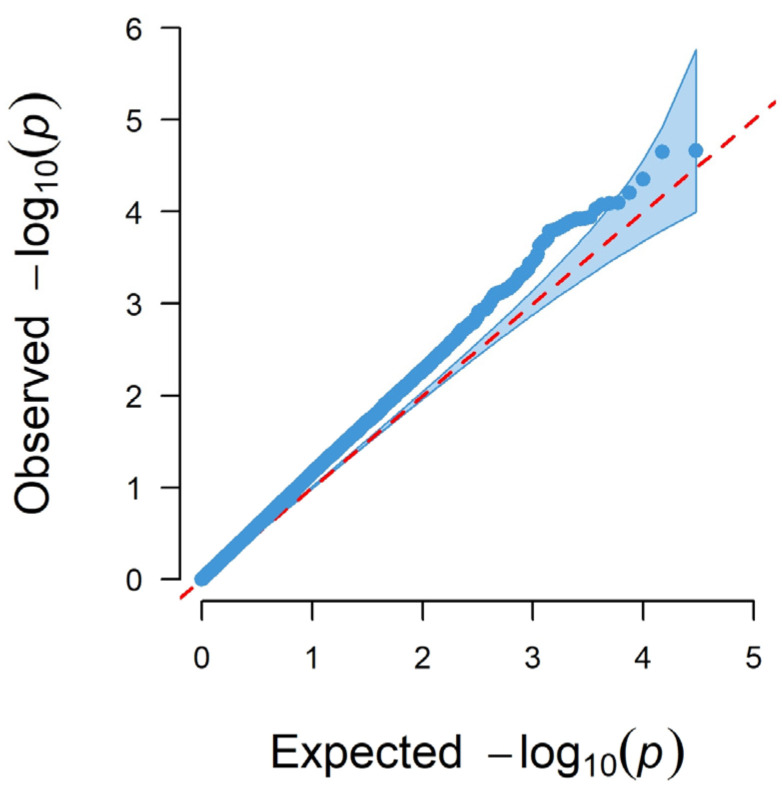
QQ plot of observed −log_10_ (*p*-values) (blue dots) and expected −log_10_ (*p*-values) (red dashed line) from GWAS results using the 50K SNP chip.

Figure 6 shows the Manhattan plot for logFEC using the 50K SNP chip and its corresponding QQ plot (Figure 7). In this case, 2203 SNPs were identified with *p*-values < 0.05, and from these, five SNPs were significant with a 5% FDR per chromosome, all of them located in chromosome 7 (Table 5). The QQ plot shows that most *p*-values follow a uniform distribution (straight line), but these five SNPs observed *p*-values deviate from the expected ones, which suggests an association between these SNPs and logFEC (Figure 7).

In OAR7, four significant SNPs are within the *INO80* gene (*INO80 complex ATPase subunit*), *TLN2* gene (*talin 2*), *TSHR* gene (*thyroid stimulating hormone receptor*) and *EIF2AK4* gene (*eukaryotic translation initiation factor 2 α kinase 4*).

### 3.2. Gene Annotation

**Figure 8:**
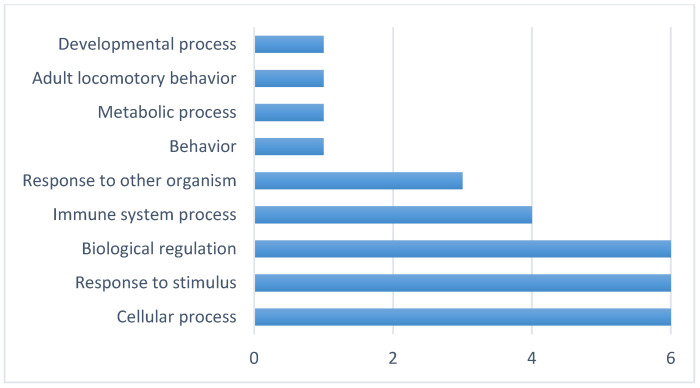
Total number of biological processes of candidate genes identified with GWAS for logFEC using 170 SNPs and the 507 and 50K SNP chips.

**Figure 9:**
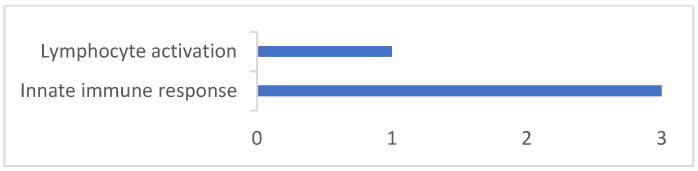
Total number of immune system processes of candidate genes identified with GWAS for logFEC using 170 SNPs and the 507 and 50K SNP chips.

After identifying the candidate genes, proteins and gene ontology (GO), terms were assigned (Supplementary Table S1). In addition, associated biological processes were identified (Figure 8) and within them, genes related to immune system processes (Figure 9). Since the sheep genome is not completely annotated, genes *SYNDIG1L*, *MGRN1*, *INO80* and *TLN2* were not mapped in the UniProt database.

## 4. Discussion

GIN infections have a significant impact on health and productivity in sheep production systems [10]. With the increasing development of anthelmintic resistance, more sustainable strategies have been sought for the control of GIN. One of these alternatives is the selection of genetically resistant animals that takes advantage of host natural immunity. Factors that affect the immune-response development rate depend on the nematode species, host breed and intensity of infection [10]. Traditional QTL mapping and GWAS studies suggest that the three mechanisms involved in host resistance to GIN are: innate and acquired immune responses to trigger T helper type 2 cytokines production (Th2); homeostatic metabolic pathways necessary for blood coagulation at the host–parasite attachment site that block parasite feeding; and gastric mucosal protection through mucins biosynthesis that acts as a barrier and trigger parasite expulsion [24]. These studies reviewed by Benavides et al. [24] suggest that GIN resistance is a quantitative trait, influenced by many genes with small effect.

In the current study, QTL associated with logFEC were explored using low density (170 and 507 SNPs) and medium density coverage (50K SNPs), with a minimum SNP overlapping only between the 507 SNP and 50K SNP chips.

By using the ssGWAS methodology, large phenotypic and pedigree databases of genotyped and non-genotyped animals could have contributed to a more reliable QTL detection [45]. In addition, as ssGWAS accounts for population structure through the relationship matrices; it reduces the probability of spurious signals [53]. In agreement with this, the QQ plots in all three SNP chips did not show large deviations from the null hypothesis and no more associations than expected by chance were observed at low levels of significance (Figure 3, Figure 5 and Figure 7).

The 170 SNPs considered here were selected in a study conducted by Periasamy et al. [26] due to their association with 76 candidate genes related to GIN infection immune response. In the current study, seven SNPs (TIMP_716, TLR5_2276, TLR5_786, LEPR_260, TLR5_2037, TLR9_2099 and TLR9_2504) in chromosomes 1, 3, 12 and 19 were identified as significantly associated with LogFEC under natural GIN infestation. The TIMP_716 SNP is located within the *metallopeptidase 3 inhibitor* gene (*TIMP3*), which codes for a group of inhibitor proteins that control extracellular matrix metalloproteases enzymatic activity (MMPs). These MMPs regulate degradation of all the extracellular matrix components and participate in the processing of bioactive molecules such as cytokines, chemokines, growth factors and their receptors [54], so they participate in different physiological processes such as innate and acquired immunity and inflammation [55]. Previous studies suggest that both MMP-2 and MMP-9 are mediators of tissue damage; the first ones maintain the intestine barrier function and the second ones participate in colitis [56]. In an experimental infection of sheep with or without previous exposure to *T. circumcincta* parasite, it was found that the last ones presented elevated levels of MMP-7 and TIMP-1 transcripts, which could be associated with a greater tissular damage in the abomasal mucosa of animals that still have not developed immunity [57]. Furthermore, differences in the expression of MMP family genes were found in the abomasal mucosa and the abomasal lymph node from resistant and susceptible sheep [58]. In agreement with Raschia et al. [52], a significant association was found between logFEC and TIMP3_716 SNP, although in different breeds (Corriedale vs. Pampinta). Pampinta is a synthetic breed composed of ¾ East Fresian and ¼ Corriedale. Raschia et al. [48] suggested that this could be due to the role that *metallopeptidase 3 inhibitor* exerts in MMPs regulation involved in tissular damage triggered by a parasitic infection.

In addition, another six significant SNPs were identified with the 170 SNPs (TLR5_2276, TLR5_786, LEPR_260, TLR5_2037, TLR9_2099 and TLR9_2504). These SNPs are located within three candidate genes (*TLR5*, *LEPR* and *TLR9*). *TLR5* and *TLR9* are genes that code for toll-like receptors (TLR), a family of transmembrane proteins present in epithelial cells that recognize molecular patterns associated with pathogens in the gastrointestinal mucosa and activate the innate immune system [59,60]. The host defense in the presence of an *H. contortus* infection has been associated with the activation of genes that code for this type of receptors [61]. In a study of resistant and susceptible Merino sheep artificially infected with *H. contortus* and *T. colubriformis*, higher expressions of *TLR2*, *TLR4*, *TLR8*, *TLR9* and *TLR10* genes were found in the abomasal mucosa of resistant animals, which could be an indication of an early inflammatory response to infection [62]. Also, in similar studies with *H. contortus* artificial infections, a higher expression of the *TLR2* gene was observed in abomasal tissue of Morada Nova-resistant lambs [61]. The significant association between logFEC and the TLR family was also described by Raschia et al. [52]. In that study, one SNP was found located within the *TLR10* gene (TLR10_292 SNP), while in the current study five SNPs were found but in the *TLR5* and *TLR9* genes instead, also in Corriedale sheep but under natural infection. This validates the relationship found in previous studies between these receptors and the immune response triggered in the host in the face of a parasitic infection.

The last significant SNP found with the 170 SNPs was the LEPR_260 that is located within the *LEPR* gene, which codes for the leptin receptor (OB receptor). Leptin is a protein that is produced in the adipose tissue, in response to feed intake and energy balance regulation [63]. Since leptin receptors and cytokine IL-6 receptors have a similar structure, leptin acts as a cytokine, also called adipokine, and participates in the innate and acquired immunity regulation [63]. Leptin levels usually increase during an infection [64] and this increase could be related to the anorexia induced during a parasitic infection [65]. A previous study by Valderrábano et al. [66] suggested that differences in immune response in pregnant ewes artificially infected with *H. contortus* could be associated with serum leptin levels. The significant association found in the current study between logFEC and LEPR_260 in the Corriedale breed validates previous studies conducted in other breeds of leptin’s involvement with the immune response in the presence of a parasitic infection.

Regarding the 507 SNP chip, in the current study significant associations were found between the logFEC and four SNPs in chromosomes 7, 12 and 24, but only two of them were within or at short distance of genes (s45225.1 and s68231.1). This last SNP is within the *mahogunin ring finger 1* gene (*MGRN1*) and the s45225.1 SNP is at 11,375 bases distance from the *synapse differentiation inducing 1-like* gene (*SYNDIG1L*). This gene codes for an integral membrane protein, which in previous studies was reported as associated with traits related to teat number in pigs [67] and to the number of thoracic vertebrae in sheep [68]. The *MGRN1* gene codes for the E3 ubiquitin ligase that participates in metallic ions union. In a GWAS for resistance to GIN in Tunisian sheep, no association was found between this gene with logFEC, but instead it was related to carcass bone weight [69]. Other studies showed this gene was associated with meat tenderness in Nellore cattle [70] and bull fertility in Holstein cattle [71].

Finally, with the 50K SNP chip significant associations were found between logFEC and five SNPs in chromosome 7, but only four of them (OAR7_37789204.1, OAR7_49479344.1, OAR7_97127242.1 and OAR7_36815076.1) were located within previously reported genes (*INO80*, *TLN2*, *TSHR* and *EIF2AK4*, respectively). *INO80* gene codes for a subunit of the INO80 chromatin remodeling complex. This complex participates in the regulation of transcription, DNA damage repair and DNA replication, processes needed for cell integrity [72]. Zhou et al. [73] found that this gene is related to oncogenic transcription and tumor growth in melanoma. The *talin 2* gene (*TLN2*) codes for a cytoskeleton protein that participates in cellular adhesion, and it was found to be associated with Alzheimer´s disease [74]. The *eukaryotic translation initiation factor 2 α kinase 4* gene (*EIF2AK4*) codes for a member of a family of kinases that regulates protein synthesis, and mutations in this gene seem to cause pulmonary veno-occlusive disease [75]. The *TSHR* gene codes for the thyroid stimulating hormone receptor that controls the thyroid cellular metabolism. Cun et al. [76] found an association between this gene and litter size in sheep from six different breeds and in another study this gene was found associated with an autoimmune disease that affects the thyroid gland (Grave’s disease) [77]. To our knowledge, none of the candidate genes found with the 507 and 50K SNP arrays have been previously reported in association with GIN resistance.

The gene ontology analysis with UniProt database showed the biological processes related to six genes found in the current study; the other four were not mapped in this database (*SYNDIG1L, MGRN1*, *INO80* and *TLN2*). The *TIMP3*, *TLR5*, *TLR9*, *LEPR*, *TSHR* and *EIF2AK4* genes are related to cellular processes (GO: 0009987), response to stimulus (GO:0050896) and biological regulation (GO:0065007), and from these only three are listed as related to immune system processes (GO:0002376; *TLR5*, *TLR9*, *TSHR*). From these three genes, two are related to the innate immune response (GO:0045087; *TLR5* and *TLR9*) and the *TSHR* gene is related to lymphocyte activation (GO:0046649) and specifically to B cell differentiation (GO:0030183).

In summary, different numbers of SNPs were statistically significant in the three panels, mainly due to the origin of the SNPs in each one, with a minimum SNP overlapping among them. The largest number of significant SNPs was found in the panel with lowest density because these SNPs were chosen based on a previous study using the candidate gene approach [26]. Our results validated the association of seven SNPs with FEC and confirm clear potential genes. In the case of the 507 SNP chip, the four significant SNPs detected were among the 91 SNPs shared with the 50K SNP panel, but they were not the same SNPs that we detected with the 50K SNP chip. Future larger training populations in the Corriedale breed would contribute to augmenting the identification of QTL associated with FEC, particularly with the 50K chip.

The variation in the SNPs and candidate genes detected with the three SNP densities confirms that GIN resistance is a complex trait influenced by many genes with small effect.

## 5. Conclusions

In the current study, genomic regions associated with GIN resistance, as well as candidate genes, were identified. Some of these candidate genes are related to the immune system (*TIMP3*, *TLR5*, *TLR9* and *LEPR*), confirming previous findings in other breeds. On the other hand, some non-reported candidate genes were found in association with logFEC, which could be valuable for future studies (*SYNDIG1L*, *MGRN1*, *INO80*, *TLN2*, *TSHR* and *EIF2AK4*). 

## Figures and Tables

**Table 1:** Descriptive statistics for animal age at recording (days), faecal egg counts (FEC) and LogFEC for the total database used (*n* = 19,547).

Trait	Mean	SD ^a^	Min ^b^	Max ^c^
Age at recording (days)	278.04	68.71	101	460
FEC	1309.98	2157.78	0	37,400
LogFEC	6.49	1.24	4.61	10.53

^a^ SD: standard deviation, ^b^ Min: minimum, ^c^ Max: maximum.

**Table 2:** Number of SNPs and animals after quality control for 170, 507 and 50K SNPs.

Density	SNP	Animals
170	148	454
507	373	702
50K	29,832	375

**Table 3:** Significant SNPs identified with GWAS for logFEC using 170 SNPs with a chromosome-wise FDR of 5%.

SNP Name	rs Code ^c^	Variant Type	*p*-Value	FDR	Chr	Position (bp) ^a^	Candidate Gene ^b^
TIMP3_716	rs159882061	downstream gene variant	0.0001	0.0056	3	176,291,630	*TIMP3*
TLR5_2276	rs429546187	missense variant	0.0012	0.0096	12	24,624,977	*TLR5*
TLR5_786	rs423611614	synonymous variant	0.0026	0.0096	12	24,626,347	*TLR5*
LEPR_260	rs416296450	intron variant	0.0026	0.0079	1	40,732,375	*LEPR*
TLR5_2037	rs410008645	synonymous variant	0.0036	0.0096	12	24,625,096	*TLR5*
TLR9_2099	rs119102850	synonymous variant	0.0045	0.0229	19	48,656,461	*TLR9*
TLR9_2504	rs119102857	synonymous variant	0.0076	0.0229	19	48,656,866	*TLR9*

^a^ Oarv_4.0, ^b^ Within 20 Kbp of marker, ^c^ [52]

**Table 4:** Significant SNPs identified with GWAS for logFEC using the 507 SNP chip with a chromosome-wise FDR of 5%.

SNP Name	rs Code	Variant Type	*p*-Value	FDR	Chr	Position (bp) ^a^	Candidate Gene ^b^
OAR12_7879376.1	rs414871182	intergenic variant	0.00124	0.0161	12	6,202,760	
s45225.1	rs402818177	intergenic variant	0.00200	0.0280	7	82,587,686	+11,375 bp of *SYNDIG1L*
s68231.1	rs410292582	intron variant	0.00551	0.0386	24	3,791,887	*MGRN1*
OAR7_50322674.1	rs427377192	intergenic variant	0.00584	0.0409	7	45,569,488	

^a^ Oarv_4.0, ^b^ Within 20 Kbp of marker.

**Table 5:** Significant SNPs identified with GWAS for logFEC using the 50K SNP chip with a chromosome-wise FDR of 5%.

SNP Name	rs Code	Variant Type	*p*-Value	FDR	Chr	Position (bp) ^a^	Candidate Gene ^b^
OAR7_37789204.1	rs426205150	intron variant	0.00002	0.0145	7	33,565,208	*INO80*
OAR7_50006482.1	rs403279855	intergenic variant	0.00002	0.0145	7	45,244,213	
OAR7_49479344.1	rs421671708	intron variant	0.00008	0.0351	7	44,708,294	*TLN2*
OAR7_97127242.1	rs412670683	intron variant	0.00012	0.0388	7	89,202,663	*TSHR*
OAR7_36815076.1	rs407390907	intron variant	0.00016	0.0405	7	32,616,683	*EIF2AK4*

^a^ Oarv_4.0, ^b^ Within 20 Kbp of marker.

## Data Availability

Restrictions apply to the availability of these data. Data were obtained by INIA and are available from the authors with INIA’s permission.

## Supplementary Materials

The following supporting information can be downloaded at: https://www.mdpi.com/article/10.3390/genes13091548/s1, Table S1: Proteins and GO terms; Table S2: Genomic estimated breeding value (GEBV) mean accuracies, standard deviations (SD), minimum (Min) and maximum (Max) using 170, 507 and 50K SNPs.
